# Ritual Slaughter as Overlooked Risk Factor for Brucellosis

**DOI:** 10.3201/eid2204.151192

**Published:** 2016-04

**Authors:** Inbal Fuchs, Lidia Osyntsov, Yael Refaely, Pnina Ciobotaro, Oren Zimhony

**Affiliations:** Clalit Health Services, Beer Sheva, Israel (I. Fuchs);; Ben-Gurion University of the Negev, Beer Sheva (I. Fuchs):; Soroka University Medical Center, Beer Sheva (L. Osyntsov, Y. Refaely);; Kaplan Medical Center, Rehovot, Israel (P. Ciobotaro, O. Zimhony)

**Keywords:** brucellosis, slaughter, respiratory infections, bacteria

**To the Editor**: The current rates of animal and human brucellosis in southern Israel are unacceptably high (*1*). Unsupervised livestock rearing, smuggling of herds, and the dissolution of Israel’s “test, slaughter, and compensate” program for small ruminants in 1997 have generated a large, uncontrolled animal reservoir in the region. The Bedouin Arab inhabitants who live in close proximity to herds and consume unpasteurized dairy products are disproportionately affected. Outbreaks from parts of Israel that were previously free of brucellosis are reported with increasing frequency. Moreover, 2 decades after supposed elimination of bovine brucellosis, a cattle herd adjacent to Bedouin grazing areas was found to be highly infected with *Brucella melitensis* (*2*).

We report 5 cases of severe brucellosis in patients from a community whose members typically do not raise herds and do not consume unpasteurized dairy products. The patients were Ethiopian-born Jews who exhibited fever and either respiratory signs or radiologic evidence of new pulmonary findings (Table). The true nature of the infection only became evident when *B. melitensis* was identified from blood cultures. Directed questioning revealed that all patients participated in ceremonial slaughter of sheep that were purchased from Bedouin owners in southern Israel.

**Table:** Characteristics, treatment, and outcomes of patients with brucellosis who engaged in ritual slaughter, Israel*Pt no.

	Age, y/sex	Clinical features	Laboratory findings at admission	Chest imaging	Alternative diagnoses	Fever-to- diagnosis interval, d	Complication	Treatment and outcome
1	68/M	Cervical neck pain; cough; night sweats; 38°C	Hb, 11.9 mg/dL; leukocytes, 16.3 × 10^3^ /μL; AST, 63 U/L; ALT, 118 U/L	CT: apical lung finding, new onset	Asthma exacerbation; lung malignancy	21	Focal lung lesion; relapse: epididymo-orchitis; suspected osteomyelitis C6: increased uptake bone scan	STR/2 wk, dox + cipro/6 wk; relapse: rising *Brucella* titers + epididymitis; same 3 drugs/12 wk; recovery
2	70/M	1st admission: fever; productive cough	Hb,14.9 mg/dL; leukocytes, 12 x 10^3^ cells/μL; platelets, 136 x 10^3^/μL; AST, 61 U/L; ALT, 47 U/L	Chest radiograph: retrocardial infiltrate	Asthma exacerbation; bronchitis	NA		Inhalatants: IV solumedrol, then oral prednisone
		2nd admission: hypothermia: 35.7°C; pulse oximetry, 94% on room air	Hb: 12 mg/dL; leukocytes, 2.8 x 10^3^ cells/ μL, 3.7 x 10^3^ cells/μL; platelets, 38 x 10^3^/μL; Na, 129 meq/L; AST, 134 U/L; ALT, 100 U/L	CT: multiple RUL pulmonary nodules; mediastinal lymphadenopathy	TB	92	Sepsis	STR/2 wk, dox/6 wk: recovery
3	45/M	Fever; prolonged headache	Hb, 11.7 mg/dL; AST, 58 U/L; ALT, 102 U/L; Na, 133 meq/L; ESR, 70 mm Hg/h	Chest radiograph: diffuse bilateral pulmonary nodules rule out miliary TB	TB; cryptococcal meningitis	21	Suspected discitis C5–6 per MRI	Genta/wk, dox + cotrim/12 wk; recovery
4	55/M	Cough; fever; low back pain	Hb, 11.8 mg/dL; AST, 86 U/L; ALT, 120 U/L; ESR, 90 mm Hg/h; CRP, 92.6 mg/L	Chest radiograph: peribronchial thickening	Pneumonia (rx cefuroxime); temporal arteritis	28		Genta/2 wk, dox/6 wk; persistent low back pain
5	49/F	Cough; fever	Hb, 9.5 mg/dL; leukocytes, 3.7 x 10^3^ cells/μL; platelets, 116 x 10^3^/μL	Chest radiograph: no pathologic changes; CT: no pathologic changes	TB; infective endocarditis caused by *Actinobacillus ureae*	90		Genta/2 wk; dox + rif/7 wk; recovery

*Pt, patient; lab, laboratory; Hb, hemoglobin; leukocyte: leukocytes; AST, aspartate aminotransferase; ALT, alanine aminotransferase; CT, computed tomographic scan; C6, cervical vertebra 6; STR, streptomycin; dox, doxycycline; cipro, ciprofloxacin; NA, not available; IV, intravenous; Na, sodium; RUL, right upper lobe; TB, tuberculosis; ESR, erythrocyte sedimentation rate; MRI, magnetic resonance imaging; genta, gentamicin; cotrim, cotrimoxazole; CRP, C-reactive protein; rx, prescription; rif, rifampin.

The diagnostic pitfalls encountered by the medical staff are exemplified in the case of patient 1 (Table). In May 2014, this 68-year-old man was admitted to a hospital in southern Israel, with a 14-day history of fever, cough, and night sweats. His medical history was notable for asthma and previously treated pulmonary tuberculosis (TB). Computed tomography scan of his chest showed a new 1.5-cm^2^ apical lung lesion with irregular borders. Laboratory evaluations were negative for rickettsiae, *Coxiella burnetii*, HIV, *Plasmodium* spp., and *Mycobacterium tuberculosis*. Four days after admission, gram-negative coccobacilli grew from his blood culture. *Brucella* IgM titer was 1:1,920. The patient received appropriate treatment for 6 weeks (Table) and recovered. *Brucella* IgM titers dropped to 1:40. Four months later, he returned to the infectious diseases clinic exhibiting fever, chills, and clinical and sonographic evidence of epididymo-orchitis. *Brucella* IgM titers rose to 1:1,240. Treatment was resumed for 3 months. In addition, a thoracoscopic biopsy of the lung lesion was performed to rule out malignancy. Pathologic examination of the biopsy specimen revealed a focus of fibrosis, with giant cells surrounded by lymphocytes (Technical Appendix Figure). After treatment, the patient’s symptoms resolved and titers returned to low levels.

The long symptom-to-diagnosis interval (range 21–97 days) for patients in this report is alarming (Table). Treatment delays are associated with increased focal complications and relapse rates (*3*). Further, high case-fatality rates, allegedly due to low physician awareness, were reported in a largely immigrant cohort of brucellosis patients in Germany (*4*). 

Several circumstances might have led to the failure to include brucellosis in the initial differential diagnosis for these patients, even in a disease-endemic region. First, we can assume that physicians are unfamiliar with the ceremonial slaughter central to the celebrations of Ethiopian Jews. The tradition includes slaughtering, skinning, and eviscerating a sheep, followed by mincing of the sheep meat. This venerated ritual is performed by trained members of the Ethiopian community and supervised by the spiritual leader, the *Kes* (*5*). Second, patients were consistently reluctant to disclose their participation in ceremonial slaughter to medical staff. Third, the managing physicians considered differential diagnoses for febrile respiratory illness in line with the patients’ Ethiopian origins: reactivation of TB or chronic pulmonary disease exacerbation (*6*). Patients 3 and 5 had a history of prolonged cough at admission; patient 3 had chest radiograph results suggestive of miliary TB. Finally, for patient 5, *Actinobacillus ureae* was initially but erroneously identified as the cause of bacteremia.

All patients in our study had clinical or radiologic evidence of lung involvement. Causality between exposure to *Brucella*-infected aerosols and pulmonary manifestations of brucellosis has been demonstrated in animal models. After an aerosol challenge with *B. melitensis*, animal lungs have shown perivascular inflammation as well as microgranulomas (*7*). In a study of hunters infected with *B. suis*, in which 38% had respiratory symptoms, aerosol spread or conjuctival innoculation was considered the most likely route of infection (*3*). 

Aerosol exposure during slaughter could be linked to the pulmonary manifestations of brucellosis observed in these patients. The granulomatous changes in the lung biopsy specimens of patient 1 are typical of lung involvement in brucellosis (*8*). The patients in this report did not use protective gear during contact with animal parts, which inevitably increased their risk for infection through direct or aerosol contact (*9*).

This reports illustrates an unsuspected mode of brucellosis transmission in an area with soaring brucellosis rates: transmission from infected animals to persons clandestinely engaging in ritual slaughter; specifically, an Ethiopian Jewish community. Physicians in countries receiving immigrants should be aware of ceremonial practices that place patients at risk for zoonoses. The severe respiratory manifestations that ensued following aerosol exposure to animal blood or secretions suggest that brucellosis with pulmonary involvement after inhalation of *Brucella*-infected aerosols might be more common than previously documented.

Technical Appendix Pathologic examination results for a patient with brucellosis, Israel, 2014.
