# Supplementary material for: Ritual Slaughter as Overlooked Risk Factor for Brucellosis
Source: Emerg Infect Dis. 2016 Apr;22(4):746–8. doi: 10.3201/eid2204.151192 (PMC4806964; doi:10.3201/eid2204.151192)
Supplement: Technical Appendix — Pathologic examination results for a patient with brucellosis, Israel, 2014. [file 15-1192-Techapp-s1.pdf]

Article DOI: http://dx.doi.org/10.3201/eid2204.151192

Ritual Slaughter as Overlooked Risk Factor for Brucellosis

Technical Appendix

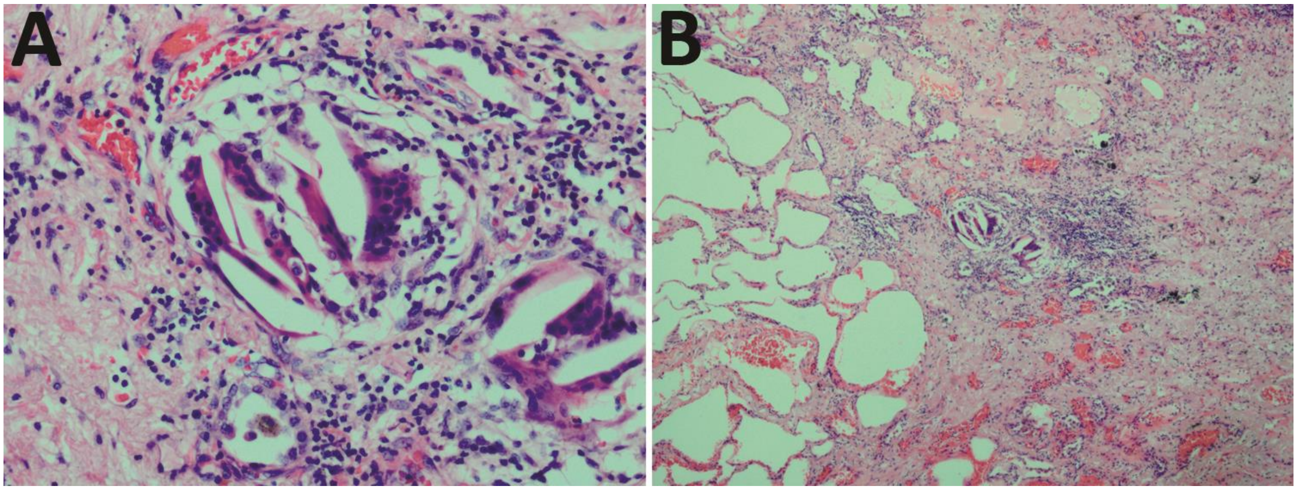

Technical Appendix Figure. Pathologic examination results for patient with brucellosis, Israel, 2014. A) Hematoxylin/eosin stain of lung tissue, with giant cells, surrounded predominantly by lymphocytes (original magnification ×200). B) Hematoxylin/eosin stain of lung tissue, showing fibrosis (right side) with a focus of giant cells, surrounded predominantly by lymphocytes (original magnification ×40).
